# Understanding the influences on hospital discharge decision-making from patient, carer and staff perspectives

**DOI:** 10.1186/s12913-024-11581-0

**Published:** 2024-09-19

**Authors:** Kristel Ward-Stockham, Olumuyiwa Omonaiye, Peteris Darzins, Clinton Kitt, Evan Newnham, Nicholas F. Taylor, Julie Considine

**Affiliations:** 1 Eastern Health, Arnold Street, Box Hill, Victoria 3128 Australia; 2 Deakin University, Geelong: School of Nursing and Midwifery and Centre for Quality and Patient Safety in the Institute for Health Transformation, 1 Gheringhap St, Geelong, VIC 3220 Australia; 3 Centre for Quality and Patient Safety – Eastern Health Partnership, Eastern Health, 5 Arnold St, Box Hill, Victoria 3128 Australia; 4 Eastern Health Institute, Eastern Health, Box Hill, Victoria 3128 Australia; 5 Eastern Health Clinical School, Monash University, Clayton, VIC 3168 Australia; 6 La Trobe University, Bundoora, VIC 3086 Australia

**Keywords:** Co-creation, Co-design, Discharge planning, General hospital, Hospital medicine, Patient-centered, Readmission, Rehospitalisation

## Abstract

**Background:**

Gaps in discharge planning are experienced by 41% of hospital patients in Australia. There is an established body of knowledge regarding the features of the discharge process that need to be improved to avoid subsequent hospital readmission and enhance the discharge experience. However, many of these studies have focused solely on factors related to unplanned hospital readmissions and there has been limited success in operationalising improvements to the discharge process. The aim of this study was to explore and describe the factors that influence the decision to discharge adult medical patients from hospital, from patient, carer and staff perspectives.

**Methods:**

A qualitative descriptive study was conducted in one acute medical ward in Melbourne, Australia. The study data were collected by observations of clinical practice and semi-structured interviews with patients, carers and staff. Participants were: i) English-speaking adults identified for discharge home, ii) patient carers, and iii) staff involved in the discharge process. Observation data were analysed using content analysis and interviews data were analysed using thematic analysis.

**Results:**

Twenty-one discharges were observed, and 65 participants were interviewed: 21 patients, two carers, and 42 staff. Most patients (76%) were identified as being ready for discharge during morning medical rounds, and 90% of discharge decisions were made collaboratively by the medical team and the patient. Carers were observed to be notified in 15 discharges by the patient (*n* = 8), doctors (*n* = 4), or nursing staff (*n* = 3). Five themes were constructed from thematic analysis of interviews: Readiness for Home, Fragmented Collaboration, Health Literacy, Unrealistic Expectations, and Care beyond Discharge. A collaborative team and supportive carers were considered to enhance risk assessment and discharge planning, however fragmented communication between clinicians, and between clinicians and patients/carers was a barrier to discharge decision-making.

**Conclusions:**

Our study highlights the need for a more coordinated approach to discharge decision-making that optimises communication with patients and carers and multidisciplinary workflows and reduces fragmentation. The importance of patient-centred care and a personalised approach to care are well established. However, there is a need to design systems to customise the entirety of the patient journey, including the approach to discharge decision making.

**Supplementary Information:**

The online version contains supplementary material available at 10.1186/s12913-024-11581-0.

## Background

Unplanned hospital readmissions are costly, distressing and inconvenient for patients and carers, increase the risk of iatrogenic harm [1], and result in potentially avoidable resource utilisation [2–4]. Lack of access to healthcare outside the hospital environment is a major factor in unplanned hospital readmissions, particularly in the first few days following hospital discharge [1, 5, 6]. Carers, who play a vital role in safe discharge and patient support at home, are often not included in discharge planning conversations or decisions [1]. Further, carer inclusion in discharge planning often occurs by chance if they happened to be on the ward, or if they insisted on involvement, usually as a consequence of previous suboptimal experiences [1].

Gaps in discharge planning are experienced by 41% of acute hospital patients in Australia [7]. Despite an established body of knowledge regarding the features of the discharge process that need to be improved to avoid unplanned hospital readmission and enhance the discharge experience [1, 6, 8], there has been limited success in operationalising improvements to the discharge process in a way that meets patient, carer and staff needs [9]. Thus, it is important to understand the factors related to discharge decision-making from patient, carer and staff perspectives as a foundation for improving the discharge process.

### Aim

The aim of this study was to explore and describe the factors that influence the decision to discharge adult patients from a hospital medical ward, from patient, carer, and staff perspectives. For the purpose of this study, carer refers to family members, or any other persons significant to the patient.

## Method

In this qualitative descriptive study, data were collected by observations of clinical practice and semi-structured interviews with patients, carers and staff. This study is reported according to the Consolidated Criteria for Reporting Qualitative Research [10]. The first three steps of the conceptual framework Functional Resonance Analysis Method (FRAM) [11] were used to guide the study conduct: i) deciding the purpose of the FRAM analysis (hospital discharge); ii) identifying the functions necessary for that work to be achieved (as defined by the participants involved in the activity) and describing each function in terms of six aspects (output, input, precondition, resource, control, and time); and iii) identify and describe variability in the identified functions.

### Ethics approval and consent to participate

This study was undertaken according to the Declaration of Helsinki [12] and was approved by the Human Research Ethics Committees at Eastern Health (LR21-019-73462) and Deakin University (2021–237). All participants provided written informed consent.

### Setting and participants

The study was conducted at Eastern Health, in Melbourne, Australia. Eastern Health provides care to 1.3 million patients per year across a large range of services and has seven hospitals. This study was conducted on a 28-bed general medical ward in a 155-bed outer metropolitan hospital. The study ward was purposively chosen for its high rate of daily discharges. The model of care on this ward is supported by daily consultant medical officer ward rounds, ward-based junior medical and allied health staff, and daily multidisciplinary team meetings. Nurse to patient ratios were 1:5 on morning, 1:6 on afternoon and 1:10 on night shift plus the nurse-in-charge. Study participants were adults (aged ≥ 18 years) who were identified for discharge and were going to their own homes. Eligible participants were identified by the nurse-in-charge, the daily multidisciplinary meeting or the electronic bed management system. Patients discharged to other facilities were excluded. Written informed consent was obtained from patients and carers for the observations of clinical practice, and from all participants who were interviewed. An opt-out consent process was used for staff who were observed. The consent process is summarised in Table 1.

**Table 1:** Consent process

	Observation of discharge process	Follow-up interview
Patients able to consent	Consent for self^*^	Consent for self^*^
Carers able to consent (if patient was agreeable to carer participation)	Consent for self^*^	Consent for self^*^
Patients unable to consent	Consent sought from carer^*^ (next of kin / guardian / authorised person)	Carer interviewed if consenting^*^
Staff (nursing, medicine, allied health clinicians, pharmacists, ward clerks)	Opt out consent	Consent for self^*^

^*^written informed consent

### Data collection and procedure

For consenting patients (self or carer), the discharge process was observed (see Supplementary Table 1 for observation schedule). The observation schedule was informed by the FRAM [11] and the interview guide was based on a review of the literature and previous work by the research team [1, 5, 6]. Patients, carers, and staff who were observed were invited to participate in a 15 to30-minute follow-up interview (see Supplementary Tables 2 and 3 for interview guides). Non-participant structured observations and semi-structured interviews were completed by one of two researchers (OO, a male doctoral prepared public health researcher or KWS, a master’s prepared female nurse researcher) between November–December 2022. One researcher (KWS) was known to ward nursing staff, neither researcher had a relationship with patients or carers, or had line management or patient care responsibilities on the study ward. Patients and carers were interviewed on the ward, in the transit lounge, or by telephone. Staff were interviewed as close to the time of discharge as possible or prior to the end of their shift on the ward. The interviews, which were audio-recorded, were a maximum of 26 min duration (average = 16.7 min). Data saturation was reached when the research team determined that no new information was coming from the observations, and the interview content was repetitive.

The tenets of rigor of qualitative research are credibility, transferability, dependability, and confirmability leading to trustworthiness [13]. Credibility was established by the systematic development of the interview guides, and the sound methodological approach ensured dependability. Confirmability was established by using examples from the observations and interviews to ensure that patients’, carers’ and staff voices were represented. A reflexive approach to thematic analysis requires researchers to question their assumptions, highlights researchers’ skills as resources, and requires researchers’ reflexive engagement with the data in its interpretation [14]. The research team was diverse and comprised researchers from nursing, medical and allied health backgrounds.

### Data analysis

The hand-written field notes from the structured observations were transcribed by the researchers (OO and KWS) and analysed using content analysis [15]. The semi-structured interviews were professionally transcribed verbatim and analysed using an inductive thematic analysis framework [14, 16]: familiarisation with the data; generating initial codes; searching for themes; reviewing themes; defining and naming themes; and producing the report. Two researchers (OO and KWS) checked the transcripts for accuracy against the audio files, entered them into NVIVO software and independently performed the initial coding. Key words and phrases were utilised to extract themes that could be used to understand participant values, attitudes, and opinions. An open coding process was used, so codes were not determined a priori, but developed and modified during the coding process [14, 16]. Two researchers (OO and KWS) individually coded the data in NVivo [17] and then came together to review the codes, and through an iterative process, identified subthemes and themes. The themes, subthemes and codes were circulated to the research team. Example interview data and their respective codes, subthemes and themes are shown in Table 2.

**Table 2:** Examples of codes, subthemes and themes

Data	Code	Subtheme	Theme
*“We think about primarily the medical—their clinical status … the patient’s symptoms. As long as they have improved” (Doctor 1).*	Medical stability	Medical stability	Readiness for home
*“I consider how I feel about them. What I see, the changes they’ve had. … I want to make sure that they’re going home and they can look after themselves” (Nurse 1)*	Improving patient safety – multidisciplinary team	Risk assessment	Readiness for home
“…*not a lot I can do at home … but … least you’re in your own environment and the dog will be happy to see me” (Patient 1).*	What matters to me – the patients	‘What matters to me’	Readiness for home

## Results

### Observations of clinical practice

Twenty-one discharges were observed. Patients’ average age was 67 years, 11 identified as female, and patients had an average hospital stay of 3.6 days. The majority of patients (*n* = 16) were identified as ready for discharge during morning medical rounds and 90% of discharge decisions were made collaboratively by the medical team and the patients. Confirmed discharges were discussed after medical rounds in the multidisciplinary meeting prior to actual discharge (*n* = 18). Patients were informed of their discharge by the medical team on morning rounds (*n* = 15) or by a member of the multidisciplinary team following the morning meeting (*n* = 6). One carer was observed to be consulted via telephone regarding readiness for discharge. Carers were notified of patient discharge by the patient (*n* = 8) or medical  (*n* = 4) or nursing staff (*n* = 3): in the other six discharges, carer engagement was not observed. Carers were the main method of transport home (*n* = 15), while four patients took taxi or ride-share at their own expense. The transit lounge was used in 14 discharges.

Patients were advised by the medical team to follow-up with their general practitioner (*n* = 16), and or a medical specialist or community allied health (*n* = 7), and three patients were informed they needed to book a medical procedure. In 12 of the observed discharges, patients were given the opportunity to ask questions, and nine patients were consulted to confirm they could physically get to an appointment. Sixteen patients received a nursing discharge summary from the nurse-in-charge: eleven patients were asked if they had questions or had their understanding of the paperwork clarified. Five patients were not observed to receive information. One of the patients was observed to receive a medical summary or correspondence to their general practitioner on request.

Pharmacists were observed to counsel patients about medications (*n* = 17), provide written medication information (*n* = 8), allow for questions (*n* = 16), clarify that information was understood (*n* = 14), and bring medications to the ward (*n* = 18). Observed communication about prescriptions was verbal (between pharmacists and nurses) or check marks on a whiteboard at the nurses’ station. Discharges identified on the day or previous day were observed to not have prescriptions written until the afternoon of the day of discharge. Awaiting prescriptions, and supply of medications was observed to delay discharges (*n* = 12). When there were time constraints such as arrival of available transport, pharmacists had less time to devote to patients and the patients were not able to ask questions.

### Interviews with patients, carers and staff

**Fig. 1:**
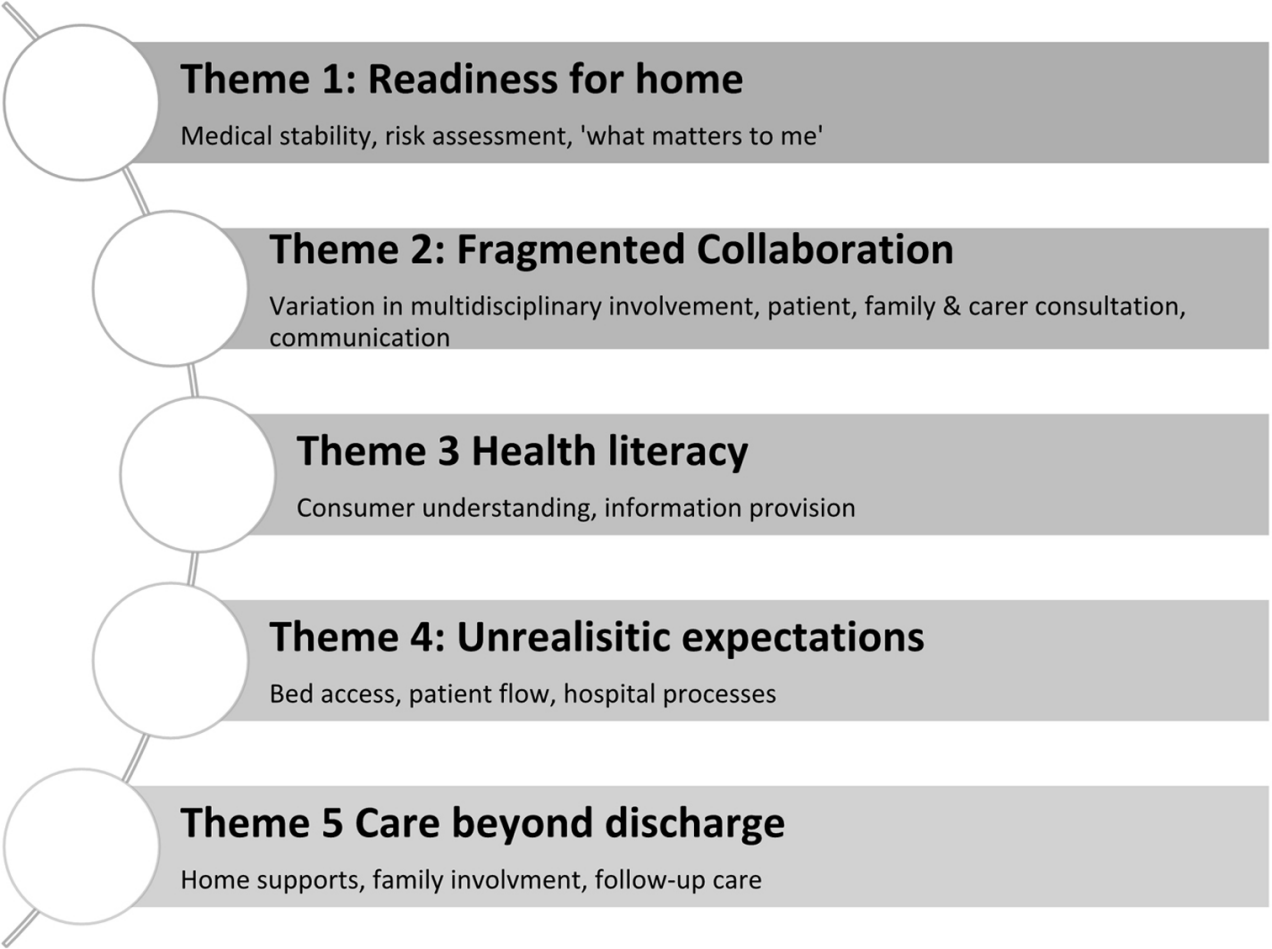
Factors influencing discharge decision making

Thematic analysis of 65 interviews from 42 staff (17 nurses, 10 doctors, 7 pharmacists, 3 ward clerks, 2 physiotherapists, 2 occupational therapists and 1 social worker), 21 patients and two carers (one adult child and one parent) resulted in five themes: Readiness for Home, Fragmented Collaboration, Health Literacy, Unrealistic Expectations, and Care beyond Discharge (Fig. 1).

#### Theme 1: Readiness for home

The first theme “Readiness for Home” was related to ensuring patients are ready for discharge using a holistic view from the healthcare professional team. Medical stability, decided by the medical consultant, was the driving force for determining discharge: *“We think about primarily the medical—their clinical status … the patient’s symptoms. As long as they have improved” (Doctor 1).* Beyond medical stability, risk factors such as functional and social concerns were considered by the nursing and multi-disciplinary teams to ensure a safe discharge and avoid readmission to hospital: “*… determining if there was any risks to this patient’s safety”* (*Social worker 1).* Medical teams relied on allied health professionals to assist in functional assessment: *“When I was going through medical training, I think talking about function is something that we mention briefly but we never focus on because our job is the medical” (Doctor 2).*

Nursing and allied health staff played a significant role in risk assessment. Nursing staff were consulted regarding the patients’ progression and reliance on nursing care: *“I consider how I feel about them. What I see, the changes they’ve had. … I want to make sure that they’re going home and they can look after themselves” (Nurse 1).* Information about function and nursing care requirements was provided to the team by the nurse-in-charge during the multidisciplinary team meeting: *“So that information would then get handed over eventually to the nurse-in-charge. She will be the one on behalf of the nurse – their assigned nurse—to relay that information to the rest of the multidiscipline team” (Nurse 2)*. Nurses expressed they could advocate for patients who were not yet ready for discharge, functionally or socially, once identified as medically stable: *“… if you think it’s not safe for someone to go home for a particular reason at that time, then we would advocate for the patient” (Nurse 3).*

Patients described the hospital environment as disruptive and noisy, and were concerned about being unable to sleep, exercise, or have their carers visit. Patients were eager to go back to their home environment: “…*not a lot I can do at home … but … least you’re in your own environment and the dog will be happy to see me” (Patient 1)*. Carers were keen to have patients at home and happy to support them in their recovery, but expressed concerns about a lack of communication regarding patients’ health status: “*I think in some ways it would be good if the hospital were able to liaise with family and fill—let us know what’s going on” (Carer 1)*.

#### Theme 2: Fragmented collaboration

The second theme “Fragmented Collaboration” describes variability in collective decision-making process, that involved to greater or lesser extents, patients, carers and the healthcare team. Medical, nursing, social work, occupational therapy, and physiotherapy staff attend daily multidisciplinary team meetings that focused on facilitating a safe discharge using a patient-centered approach. The opportunity to communicate and share decisions within the multidisciplinary team was a facilitator to assessing risks and identifying barriers to discharge. Staff expressed that the multidisciplinary team meeting was a facilitator to collaborative decision-making and communication:

*“…having a chance for the nursing staff, the physios and the OTs to sit down, and the medical staff, is always good … that discussion at the 11 o’clock meeting is always a good way of talking about discharge destinations”. (Doctor 3)*

Nurses felt they should have more input into discharge decisions: “*The nurses looking after the patient should definitely be involved in the discharge” (Nurse 4).* However, nurses also reported being involved in decision-making only if they were in the room at the time of discussion: *“… it’s when we’re in – we’re busy and we’re in a hurry and the doctors have made decisions without consulting us” (Nurse 1).*

Decisions about medical stability for discharge were made by the medical team, and patients commented on a lack of involvement: “*I know that they’ve [doctors] made a decision and it’s impossible for you [doctors] to be in conference with your patients about the decisions that you’re making” (Patient 2).* Patients felt they had not received enough information about their health, rationale for new medication, or management of their condition:* “I’m actually going to take that script and go to my [general practitioner] GP who I trust and have a conversation with them about it” (Patient 2).*

Visitor restrictions due to the COVID-19 pandemic meant that carers had little opportunity to contribute to the discharge discussion in person until discharge was confirmed. When carers were asked about being notified of patients’ discharge they replied*: “…I was on the phone to the doctor this afternoon and he just notified …she [the patient] can go home. So, I said, just give me a couple of hours and he said, how about four o’clock?” (Carer 1).* Nurses also noticed the lack of involvement of carers: “*We have the meeting, however, things get discussed in there that they don’t seem to come out to the patient or the families out here” (Nurse 5).* Carers were contacted by the patient, the nurse-in-charge, or the medical team after the decision to discharge was made but they expressed wanting to be more involved in discharge planning: *“I would like to hear from the doctors and things, what’s happening and where she is at and what the—moving forward is going to mean and look like” (Carer 2).* Healthcare professionals recognised the importance of carer input in assessing risks and barriers to returning home, communicating social and functional issues, available supports at home, and if there were any concerns:

*“Sometimes it’s helpful to have that person there to tell us what’s actually happening from a third-person perspective, because sometimes patients don’t always tell you the truth, or they minimise because they know that it would delay discharge if they tell you they’re struggling at home” (Doctor 2).*

Communication of discharge decisions between staff was ad hoc. Clinicians’ knowledge about systems and local processes used to communicate discharge decisions was varied:

*“…if we have a page, a board maybe, about what needs to be done prior to discharge, even if it’s an electronic system that we can all tick off. We just don’t have that system.* W*e have …[electronic bed management system] but it doesn’t give me the information of what … needs to be done” (Doctor 2).*

Therefore, the nurse-in-charge assumed the critical role of coordinating the discharge process *“… the nurse in charge is the one that coordinates the discharge” (Social Worker 1)* and maintained workflow “…*it relies a lot on the nurses to keep the flow going and tell us who to see and who to discharge first … so we rely on them (Doctor 5)”.* The nurse-in-charge also facilitated communication between disciplines *“…they had to update me as well as pharmacy, doctors, interns and pharmacist” (Nurse 6),* completed paperwork, and kept the patient updated “*…once I know, I will let the patient know so then she can contact the next of kin…” (Nurse 2).*

#### Theme 3: Health literacy

The third theme “Health Literacy” refers to the knowledge and skills patients need to comprehend their own health needs. Patients identified difficulties in understanding medical information such as discharge medications:

“…*they gave me a sheet of all the medication I’m taking … they also had a printout like what they do, what each tablet does, because I wouldn’t have a clue. ... I can’t even pronounce the name, let alone know what I’m taking” (Patient 2).*

Patients described receiving verbal information from multiple sources creating confusion: *“I have had a lot … of people … but a single font of information would have been … handy just for clarity.” (Patient 2).* Staff identified that information from multiple sources was problematic *“It probably feels clunky to the patient” (Nurse 8)* and* “… the information needs to be more streamlined” (Patient 2).* Both patients and staff highlighted the need for greater coordination of information: patients felt that “*… having that singular interface, an overriding liaison …one person who would come in and say this is where you’re at …” (Patient 2)* was important and staff had similar comments* “… allocate[ing] someone to be … that person’s discharge person who’s … responsible for that communication with the patient and their carer” (Physiotherapist 1)*.

Participants identified opportunities to improve discharge documentation. For example, medical staff commented *“Give them their discharge summary from this admission just so that they have a written copy of all the issues that have happened and all the investigations that have been looked at” (Doctor 1)*. Doctors wanted to give patients their discharge summary before the patient left the ward: “…*we try to give them their discharge summary from this admission” (Doctor 1)* but reported excessive workload and time constraints resulted in* “… a backlog of them [discharge summaries]” (Doctor 3)*. Patients found discharge summaries from previous admissions to be useful: *“When I was discharged from ED [Emergency Department] I got a printout of what was sent to the doctor. That was useful, even though it was all stuff I don’t really understand” (Patient 5).* Patients found nursing documentation was less useful:

*“…the paperwork needs to be more detailed because not everyone leaving hospital is in a space to receive this information and retain it” (Patient 4).*

#### Theme 4: Unrealistic expectations

Increased patient care needs, and workforce shortages have resulted in significant workload pressures affecting staff, patients, and carers. Staff shortages affected all professions *“…if the workload is not permissible, we will skip those bits [risk assessments] and let the ward team handle it” (Doctor 2)* and *“it’s not solely clinical, we’re juggling lots of different things …” (Pharmacy 1).* Nurses described limited capacity to update patients and carers, “*… because of the workload as well. It’s usually a phone call once they’re confirmed to say yep, they’re going home” (Nurse 5)*.

Staff described feeling pressured to rush discharges to create bed capacity: *“I feel that it’s sometimes too rushed, especially when there’s pressure from the executives…” (Ward clerk 1)* and associated risks *“…things are obviously going to be missed because the pressure’s on and that’s not fair on the staff or the patient” (Ward clerk 1)*. Staff commented that patients are sometimes discharged before they are well enough “*… it’s just about addressing … why they don’t feel like they’re ready and supporting them and saying, the medical team think you’re ready” (Doctor 5)*. On the other hand participants also identified risk aversion influenced decision-making, resulting in longer lengths of stay with poorer patient outcomes:

*“I find certain doctors are too risk averse for a discharge and we should be aiming to get more people out the door… There’s a lot of information to suggest that actually staying in hospital when you don’t need to is obviously bad for you. You become deconditioned; you need rehab.” (Doctor 3)*.

Adding to the pressure of timely and safe discharges were local process barriers. Delays in discharge were often caused by waiting for prescriptions *“…so we wait for a script to be prepared and that can be sometimes hours” (Nurse 8)*, medications *“… a lot of things happen during the whole dispensing or supply of the medication” (Pharmacist 2),* or transport *“… ambulances … in some cases we’ve waited for hours” (Ward clerk 2)*.

#### Theme 5: Care beyond discharge

The final theme “Care Beyond Discharge” is about patients and carers still requiring care and support once they are at home. Nursing staff played a central role in judging home and carer supports, patients’ abilities to manage at home, and identifying when allied health staff should be involved:

*“I will ask the patient themselves, what are their concerns? … then … the appropriate profession will be notified as well so then they can prioritise coming in and answer those questions for the patient.” (Nurse 2)*.

Allied health assessments are critical to “*… make sure that the patient is well supported in the community when they go” (Doctor 1),* and allied health professionals talked about nursing and medical staff *“keep[ing] the patient until we have been able to complete our assessment, which is a good thing I guess in terms of determining risks” (Social Worker 1)*. Carers expressed their willingness to support patients at home, *“there’s a support network. myself and other adults … who can jump in and out to support her with her recovery” (Carer 1)*. Patients described challenges in accessing follow-up care: *“… the hardest part is getting into podiatry …. I was looking for like a year” (Patient 1).* Staff also expressed difficulties in organising post discharge community support:* “..they don’t have the staff to.. provide that service in the home… staff are sick” (Social Worker 1).*

Despite all patients being advised to follow-up with their general practitioner, communication with primary care providers and medical specialists was limited and clinicians expressed wanting time to contact the general practitioner prior to discharge:* “…a call to his GP [general practitioner] probably is a good idea, but … in practice, actually we don’t really call GPs that often unless it’s very complicated, just because of time demands” (Doctor 3).* The acute hospital environment, high patient turnover and time constraints of general practitioners were cited as making communication with general practitioners difficult: *“…the GP’s too busy. They have got their own things to do. So, I think the written summary is probably the best, still” (Doctor 7)*.

## Discussion

In this study we explored and described the factors that influenced discharge decision-making for adult patients with medical conditions, from patient, carer and staff perspectives. Based on observations of the discharge process, and follow-up interviews with patients, carers and staff, the major influences on discharge decision-making were: i) patient factors, ii) staff capability given various work pressures, and iii) the interplay between patients, carers and staff.

Patient factors that influenced discharge decision-making were readiness for discharge, health literacy and care beyond discharge. Patient’s clinical “readiness for discharge” was largely determined by medical staff with input from other professions commonly an afterthought or the result of opportunistic presence during medical rounds. Patients and carers in this study wanted more input into discharge decision-making and patients, carers and staff all expressed a need for greater carer involvement. Other studies have shown that patients and carers often trust healthcare professionals to make discharge decisions [18].

Patient health literacy (ability to use reading, writing, verbal, and numerical skills [19] and, or language concordant care [20]) was both observed and reported in interviews with patients / carers as an influence on discharge decision-making. Patients with limited health literacy are known to have poor health outcomes, including increased risk of unplanned emergency department visits or hospital readmission following hospital discharge [19]. One of the issues that may exacerbate limitations in health literacy for patients and carers is the volume of information received during this discharge process, which was observed in our study and reinforced by patients and carers during the interviews. Patients and carers described being overwhelmed by large quantities of verbal information coming from many different people, and that written information was not always meaningful. Other studies of patients’ perception of communication of discharge decisions also allude to a need for patient-centred communication, use of understandable language, and checking that patients and carers understood the information presented to them [21].

Many participants commented that a person acting as a single point of contact would be helpful in navigating decisions during the discharge process. The complexity of care needs, and difficulties accessing primary care or community health following discharge also added weight to the notion of a discharge coordinator. ‘Navigation’ programs or discharge coordinators improve outcomes and care experiences for patients and carers in various contexts, including hospital discharge [22, 23]. The key responsibilities of the discharge coordinator would be to address patient and family concerns, answer questions, and engage patients and families [23].

Patient care needs beyond discharge were recognised as important and also influenced discharge decision-making, but observations and interviews both showed that medical staff were less likely than nursing or allied health staff to recognise ‘non-medical’ care needs at home. The optimal model of post-discharge care is unclear for a number of reasons. First, the outcome of interest varies between studies and includes unplanned readmission avoidance [24], function and prevention of functional decline [25], and older persons experiences of adapting back to life at home after hospitalisation [26]. A meta summary of findings from 13 qualitative studies of older patients’ experience of managing at home after hospital discharge found four themes: i) experiencing an insecure and unsafe transition, ii) settling into a new situation at home, iiii) what would I do without my informal caregiver? and iv) experience of a paternalistic medical model [26]. The results of this study [26] and our study both highlight the importance of planning, information and involvement of patients and carers in decisions about discharge and follow-up care.

The staff capability factors that influenced discharge-decision-making were workflows and unrealistic expectations. Shared decision-making during discharge planning is highly valued by patients [21]. Our data showed that current workflows often precluded timely and shared discharge decision-making. Better communication between patients, carers and staff was highlighted as important in our study and has been a major finding in other studies of hospital discharge [26].

In our study, many organisational expectations were viewed by clinicians as unrealistic, of limited feasibility, or creating other pressures that hindered the decision-making process. Many organisational imperatives focus on morning discharge with the intent of improving hospital throughput and reducing emergency department overcrowding [27, 28]. The flow-on impacts to inpatient clinician roles are often neglected in these initiatives [27, 28]. Co-design with patients and carers of such initiatives is often lacking and can result in a less holistic approach to the experience of discharge for patients [28, 29].

Finally, there was a complex interplay between patients, carers and staff. Carers were rarely present during discharge decision-making (despite patients, carers and staff all expressing a desire for greater carer input). Carers tended to be engaged or informed once decisions to discharge the patients had already been made, despite carers’ important roles in confirming the patients’ baseline level of function, assisting patients at home, and bridging any gaps and providing care while patients wait for services [23]. Despite multidisciplinary team meetings being highly valued by many staff and described as facilitating risk assessment, and discharge planning, observations of clinical practice showed that discharge decisions were largely operationalised around the needs of medical staff, and notably, did not actively engage patients or carers. Other studies have reported tension at multidisciplinary team meetings, largely due to differing goals of different professional groups. For example, medical staff under pressure to send patients home focused on medical stability but nursing and allied health staff focused on whether patients were physically or cognitively safe for discharge [23]. Co-design of increasing patient or carer (who wish to be involved) involvement and engagement in multidisciplinary team meetings warrants further consideration and evaluation from patient, carer and clinician perspectives.

### Strengths and limitations

Use of a rigorous qualitative methodology with direct observations supplemented by interviews, and allowing the expression of experience, perspectives and opinions from patients, carers and staff was a strength of this study. The interviews were conducted directly after the discharge, thus reducing recall bias and enabling sharing of fresh experiences. There are limitations that should be considered when interpreting the study findings. Patients interviewed were English language speakers discharged to home and who were without cognitive impairment. Capturing the experiences of patients living in residential facilities, or with limited English proficiency should be a focus of further work. The focus of this work was patients discharged from hospital on the days of data collection and who had a predetermined decision that they were suitable for hospital discharge. Thus, patients (and carers) deemed not for hospital discharge were not included in this study. The factors that influence the decision not to discharge patients from hospital remains a knowledge gap that should be addressed in future studies. The study took place while visitor restrictions were in place due to COVID-19, thus limiting access to carers.

## Conclusion

Our study highlights gaps in the approach to discharge decision-making for patients in whom a decision to discharge has been made, which has implications for workflow, communication and patient safety. Early two-way communication by staff with patients and carers, that could enhance patients’ and carers’ appreciation of their situations is lacking. Facilitation of communication by a designated staff member may improve the quality of discharge decision-making. Medical staff have a dominant role in discharge decision-making from the acute hospital setting, with multi-disciplinary team involvement having a lesser role, yet involvement of the latter is perceived to be beneficial and is desired. These insights may inform optimisation of discharge decision-making processes. Finally, the drivers of decisions not to discharge patients from hospital are still poorly understood and should be an area for future research.

## Supplementary Information

Supplementary Material 1.

## Data Availability

The datasets used and/or analysed during the current study are available from the corresponding author on reasonable request.

## Abbreviation

FRAM: Functional Resonance Analysis Method
