# Supplementary material for: Understanding the influences on hospital discharge decision-making from patient, carer and staff perspectives
Source: BMC Health Serv Res. 2024 Sep 19;24:1097. doi: 10.1186/s12913-024-11581-0 (PMC11411756; doi:10.1186/s12913-024-11581-0)
Supplement: Supplementary file 1 — Supplementary Material 1. [file 12913_2024_11581_MOESM1_ESM.docx]

## Supplementary Table 1: Structured Observation Guide

| **Functions to achieve hospital discharge** | |
| --- | --- |
| Identifying patients who are ready for discharge | *How does this process occur? By whom? Shared decision making?* |
| Notifying patients that the patient is ready for discharge | *By whom? Shared decision making?* |
| Notifying carers that the patient is ready for discharge | *By whom? Shared decision making?* |
| Medications | *Is the patient discharged with medications? Are there new medications?*  *Does anyone explain medications to the patient / carer, particularly new medications? If yes ® whom?*  *Is the patient / carer given written information? If yes ® by whom?*  *Is the patient / carer given the opportunity to ask questions?*  *Does the clinician confirm that the patient / carer understands medication requirements?*  *Are the medications brought to the ward? If yes ® by whom? Or does the patient / carer have to collect them? If yes ® from where?*  *Are medications a source of discharge delay?* |
| Appliances and equipment (e.g. gait aids, dressings) | *Is the patient discharged with appliances / equipment? Are there new appliances / equipment?*  *Does anyone explain appliances / equipment to the patient / carer, particularly new appliances / equipment? If yes ® whom?*  *Is the patient given the opportunity to practice or test new appliances / equipment?*  *Is the patient / carer given written information? If yes ® by whom?*  *Is the patient / carer given the opportunity to ask questions?*  *Does the clinician confirm that the patient / carer understands medication requirements?*  *Are the appliances / equipment brought to the ward? If yes ® by whom? Or does the patient / carer have to collect them? If yes ® from where?*  *Are appliances / equipment a source of discharge delay?* |
| Post-discharge support services (e.g. meals of wheels, home help, district nursing) | *Is the patient assessed for post-discharge support services? If yes ® by whom?*  *Does the patient have a referral to post-discharge services? Which services? How does this process occur? By whom? Shared decision making?*  *Does anyone explain the rationale for referral and services offered? If yes* ® *by whom?*  *Is the patient / carer given written information? If yes ® by whom?*  *Is the patient / carer given the opportunity to ask questions?*  *Does the clinician confirm that the patient / carer understands whether the service will be delivered in the home or in the community?*  *If in-home service, is the patient / carer informed of who will visit and when?*  *Are referrals a source of discharge delay?* |
| Post-discharge follow-up appointments (e.g., outpatients, general practitioner, specialist, pathology, radiology) | *Does the patient have a post-discharge referral? Which services? How does this process occur? By whom? Shared decision making?*  *Does anyone explain the rationale for referral (s)? If yes* ® *by whom?*  *Is the patient / carer given written information? If yes ® by whom?*  *Is the patient / carer given the opportunity to ask questions?*  *Does the clinician confirm that the patient / carer understands where and when those appointments will be? Is there any discussion of how the patient will get to those appointments?*  *Are appointments a source of discharge delay?* |
| Documentation / correspondence (e.g., GP or specialist letter, discharge summary, sick leave / carers leave certificate) | *Is the patient / carer provided with correspondence? If yes* ® *by whom? To whom is the correspondence addressed?*  *Does the patient / carer have to ask for specific documentation / correspondence?*  *Does anyone explain the purpose and content of the correspondence? If yes* ® *whom? Shared decision making?*  *Is the patient / carer given written information? If yes ® by whom?*  *Is the patient / carer given the opportunity to ask questions?*  *Does the clinician confirm that the patient / carer understands what to do with any correspondence provided?*  *Is documentation a source of discharge delay?* |
| Transport | *How will the patient get home? Who makes this decision? Shared decision making – patient / carer?*  *Does the patient leave from the ward or via transit lounge?*  *Is there a cost to transport (e.g., taxi)?*  *Is transportation a source of discharge delay?* |

## Supplementary Table 2: Interview Guide for Patients and / or Carers

| Example questions (will be tailored to patient or carer) |
| --- |
| - Have you had many admissions to hospital in the last 6 months? - When did you find out you were going home? - How ready do you feel to go home? - What are you looking forward to the most about going home? - Do you feel like you have enough information about your condition to manage at home? - Do you feel you understand which medications you need to take at home, what they are for and when you need to take them? - How are you getting home? - Do you have someone that helps you once you get home? - Do you understand what follow-up appointments you will need to have once you go home? - Have you been told that there are activities you cannot do once you are at home? - Do you have any other comments about the process of going home from hospital? If there was one thing we could improve, what would it be? |

## Supplementary Table 3: Interview Guide for Staff

| Example questions (will be tailored to staff role) |
| --- |
| - Patient [name] was discharged [insert day]. Can you tell me how the decision for discharge was made? - Once the decision is made that someone is ready to go home, how is that information conveyed? - If a patient or carer says they are not ready for discharge, how is this managed? - Do you feel like you have a strong sense of how patients / carers will manage at home? - Do you feel that patients / carers understand the medications they need to take at home and what they are for? - Can you tell us about the process for the patient to actually leave the ward? - Do patients / carers ever contact the ward once they are at home? - In your experience, do many patients experience an unplanned hospital readmission soon after they go home? - Do you have any other comments about the process of going home from hospital? If there was one thing we could improve, what would it be? |
